# Nomogram for predicting survival of patients with metastatic nonfunctioning pancreatic neuroendocrine tumors: A SEER based study

**DOI:** 10.1097/MD.0000000000026347

**Published:** 2021-07-09

**Authors:** Lina Ge, Haijin Li, Liang Dong, Guanmin Shang, Weiying Wang, Ying Li, Liping Qi, Jiangang Zhao, Dengfu Peng, Guoqi Tong

**Affiliations:** Department of Oncology, Shaoxing Central Hospital, Shaoxing, Zhejiang, China.

**Keywords:** general surgery, nomogram, pancreatic neoplasms, the surveillance, epidemiology, and end results program

## Abstract

More attention has been placed on nonfunctioning pancreatic neuroendocrine tumors due to the increase in its incidence in recent years. Whether tumor resection at the primary site of metastatic NFpNET is effective remains controversial. Moreover, clinicians need a more precise prognostic tool to estimate the survival of these patients.

Patients with metastatic NFpNET were extracted from the Surveillance, Epidemiology, and End Results (SEER) database. Significant prognostic factors were identified using a multivariate Cox regression model and included in the nomogram. Coarsened exact matching analysis was used to balance the clinical variables between the non-surgical and surgical groups in our study.

A total of 1464 patients with metastatic nonfunctioning pancreatic neuroendocrine tumors (NFpNETs) were included in our cohort. Multivariate analysis identified age, sex, tumor size, differentiated grade, lymph node metastases, resection of primary tumors, and marital status as independent predictors of metastatic NFpNET. The nomogram showed excellent accuracy in predicting 1-, 3-, and 5-year overall survival, with a C-index of 0.812. The calibration curve revealed good consistency between the predicted and actual survival.

Coarsened exact matching analysis using SEER data indicated the survival advantages of resection of primary tumors. Our study is the first to build a nomogram model for patients with metastatic NFpNETs. This predictive tool can help clinicians identify high-risk patients and more accurately assess patient survival times.

## 1 Introduction

Pancreatic neuroendocrine tumors (pNETs) are a diverse group of rare neoplasms derived from peptide neurons and neuroendocrine cells, and comprise approximately 2% of all pancreatic neoplasms.^[1]^ However, the incidence of this disease has been increasing in the United States over the last few decades, with an estimation of 5.25 per 100,000 people.^[2]^ Pancreatic neuroendocrine tumors are typically classified as functional or nonfunctional according to their ability to secrete biologically active hormones and elicit characteristic symptomatology. Up to 75% of all pancreatic neuroendocrine tumors are nonfunctional,^[3]^ and given a delay in symptoms due to their indolent nature, the majority of patients are diagnosed at advanced stages leading to challenging medical management. Given its rarity, the clinical and pathological features of metastatic nonfunctioning pancreatic neuroendocrine tumors (NFpNETs) are still poorly defined. Considering the poor prognosis of metastatic NFpNET and inaccurate prediction of long-term survival for individual patients, a more precise prognostic tool is needed to estimate the survival of these patients to assist in clinical decision making and optimization of therapeutic strategies.

As a type of prognostic tool, the nomogram model incorporates multiple prognostic factors to provide more accurate and powerful information in the clinical setting, and this model has been applied to various types of cancers.^[4,5]^ In this study, we aimed to establish a prognostic nomogram to estimate individualized survival probabilities for patients with metastatic NFpNET. We compared the accuracy of the nomogram model with the predictive value of tumor differentiation grade.

Furthermore, the role of surgical management in patients with metastatic NFpNETs remains controversial. Studies supporting the resection of the primary site in patients with metastatic NFpNET are usually limited by selection bias. Thus, we also investigated the role of surgical intervention in metastatic NFpNET patients via the coarsened exact matching (CEM) approach.

## 2 Methods

### 2.1 Study design and patient population

The surveillance, epidemiology, and end results (SEER) database, which contains prospectively collected data on demographics, lesion, first course of treatment, and survival of all cancer patients from state cancer registries across the United States, was utilized for case extraction. The inclusion criteria were as follows:

1. diagnosed with NFpNET as defined by the International Classification of Diseases for Oncology Third Edition histology codes (8013/3, 8150/3, and 8240/3–8249/3) and site code (C25.0-C25.4 and C25.7-C25.9);
2. Patients with distant metastases according to the SEER historic stage variable or the American Joint Commission on Cancer stage IV;
3. NFpNET was the only or first malignancy;
4. Patients had completed follow-ups.

Patients with NFpNET diagnosed by autopsy or without histological confirmation were excluded from the study. Patients with unknown race, tumor size, differentiated grade, lymph node metastasis, and surgery were also excluded. The institutional review board of Shaoxing Central Hospital approved this study.

### 2.2 Definition of variables and endpoint

The clinicopathologic variables, including age at diagnosis (<60 or ≥60 years), race (white or non-white), sex (male or female), year of diagnosis, tumor differentiation grade (well-differentiated, moderately differentiated, poorly differentiated, or undifferentiated), primary site of pancreatic tumor (head, body, tail, or others), tumor size, lymph node metastases, resection of primary site, radiotherapy (yes or no/unknown), chemotherapy (yes or no/unknown), and marital status (married, single, or unknown) were extracted for our analysis. Overall survival was used as an endpoint, which was defined as the length of time in months from diagnosis to death from any cause or last follow-up.

### 2.3 Statistical analyzes

We employed univariate and multivariate Cox proportional hazards regression models to assess the prognostic value of the variables involved. Significant variables (*P* < .1) in the univariate model were further analyzed in the multivariable analysis. Based on the results of multivariate analysis, a nomogram was established to combine all the independent prognostic factors (*P* < .05) to predict the 1-, 3-, and 5-year overall survival of patients with metastatic NFpNET. The calibration curves were plotted to compare the association between nomogram-predicted overall survival (OS) and actual outcomes. The discriminative ability of the predictive model was evaluated using the concordance index and the receiver operating characteristic curve (ROC) with the area under the curve (AUC). Decision curve analyzes (DCA) were used to evaluate the clinical usefulness and benefits of the nomogram model. Bootstrap analyses with 1000 resamples were conducted for these analyzes.

Coarsened exact matching (CEM), which is able to achieve lower levels of imbalance, model dependence, and bias than propensity score matching,^[6]^ was applied for statistical matching. Survival curves were drawn using the Kaplan–Meier method. R version 3.4.5. was used for all statistical analyses. A two-sided *P* value of <.05, was considered statistically significant.

## 3 Results

### 3.1 Clinicopathologic characteristics of patients

A total of 1,464 patients diagnosed with metastatic NFpNETs were identified between 2000 and 2015. More than half (53.6%) of the patients were male, and the Caucasian race (77.0%) made up the majority. Most patients were diagnosed with a well-differentiated tumor size of >4 cm and lymph node metastases. Most patients underwent surgical treatment. The detailed demographic and clinicopathological characteristics are summarized in Table 1.

**Table 1:** Demographic, clinical, and pathologic characteristics of study cohort.

Characteristic	N (%)
All	1464 (100%)
Age group, y	
<60	755 (51.6%)
≥60	709 (48.4%)
Sex	
Female	680 (46.4%)
Male	784 (53.6%)
Race	
White	1127 (77.0%)
Non-white	337 (23.0%)
Year of diagnosis	
2000–2005	162 (11.1%)
2006–2010	385 (26.3%)
2011–2015	917 (62.6%)
Tumor location	
Head of pancreas	399 (27.3%)
Body of pancreas	212 (14.5%)
Tail of pancreas	560 (38.2%)
Other sites	293 (20.0%)
Tumor size	
<2cm	268 (18.3%)
≥2 cm and <4 cm	456 (31.1%)
≥4 cm	740 (50.5%)
Differentiated grade	
Well	854 (58.3%)
Moderate	341 (23.3%)
Poor	200 (13.7%)
Undifferentiated	69 (4.7%)
Lymph node metastases	
No	804 (54.9%)
Yes	660 (45.1%)
Operation performed	
No	444 (30.3%)
Yes	1020 (69.7%)
Radiotherapy	
No/unknown	1371 (93.6%)
Yes	93 (6.4%)
Chemotherapy	
No/unknown	1085 (74.0%)
Yes	380 (26.0%)
Marital status	
Single	487 (33.3%)
Married	909 (62.1%)
Unknown	68 (4.6%)

### 3.2 Independent prognostic factors of overall survival

We then used univariate and multivariate analyzes to evaluate the prognostic value of the included variables. Univariate analysis identified age at diagnosis, sex, year of diagnosis, tumor location, tumor size, tumor differentiation grade, lymph node metastases, surgical treatment, radiotherapy, chemotherapy, and marital status for further analysis (*P* < .1, Table 2). On multivariate analysis, we found that age at diagnosis, sex, tumor differentiation grade, lymph node metastases, and surgical treatment were independent risk factors for prognosis (*P* < .05, Table 2), whereas both tumor size (*P* = .064) and marital status (*P* = .051) had borderline significance (Table 2).

**Table 2:** Cox proportional hazards regression model for overall survival.

	Univariate		Multivariate	
Characteristic	HR (95% CI)	*P*	HR (95% CI)	*P*
Age group, y				
<60	1.00 [reference]		1.00 [reference]	
≥60	1.589 (1.341–1.884)	<.001	1.590 (1.335–1.894)	<.001
Sex				
Female	1.00 [reference]		1.00 [reference]	
Male	1.356 (1.142–1.609)	<.001	1.197 (1.000–1.432)	.050
Race				
White	1.00 [reference]			
Non-white	0.874 (0.710–1.076)	.203		
Year of diagnosis				
2000–2005	1.00 [reference]		1.00 [reference]	
2006–2010	0.818 (0.642–1.043)	.105	0.834 (0.652–1.067)	.150
2011–2015	0.644 (0.504–0.823)	<.001	0.707 (0.551–0.908)	.006
Tumor location				
Head of pancreas	1.00 [reference]		1.00 [reference]	
Body of pancreas	0.645 (0.485–0.859)	.003	0.881 (0.660–1.176)	.389
Tail of pancreas	0.663 (0.537–0.818)	<.001	0.935 (0.753–1.161)	.542
Other sites	0.981 (0.782–1.230)	.867	1.058 (0.837–1.338)	.636
Tumor size				
<2cm	1.00 [reference]		1.00 [reference]	
≥2 cm and <4 cm	2.626 (1.828–3.772)	<.001	1.327 (0.911–1.934)	.140
≥4 cm	3.603 (2.556–5.079)	<.001	1.410 (0.980–2.030)	.064
Differentiated grade				
Well	1.00 [reference]		1.00 [reference]	
Moderate	1.329 (1.058–1.670)	.015	1.271 (1.007–1.604)	.044
Poor	5.605 (4.560–6.888)	<.001	3.781 (3.004–4.759)	<.001
Undifferentiated	6.449 (4.741–8.771)	<.001	3.857 (2.783–5.346)	<.001
Lymph node metastases				
No	1.00 [reference]		1.00 [reference]	
Yes	1.249 (1.056–1.479)	.010	1.372 (1.148–1.639)	.001
Surgery				
No surgery	1.00 [reference]		1.00 [reference]	
Surgery	0.171 (0.144–0.204)	<.001	0.213 (0.173–0.263)	<.001
Radiotherapy				
No/unknown	1.00 [reference]		1.00 [reference]	
Yes	2.184 (1.669–2.858)	<.001	0.841 (0.630–1.122)	.238
Chemotherapy				
No/unknown	1.00 [reference]		1.00 [reference]	
Yes	3.320 (2.801–3.936)	<.001	1.178 (0.958–1.449)	.120
Marital status				
Single	1.00 [reference]		1.00 [reference]	
Married	0.850 (0.712–1.014)	.071	0.833 (0.694–1.001)	.051
Unknown	0.683 (0.426–1.094)	.112	0.857 (0.531–1.382)	.527

### 3.3 Prognostic nomogram for overall survival

**Figure 1:**
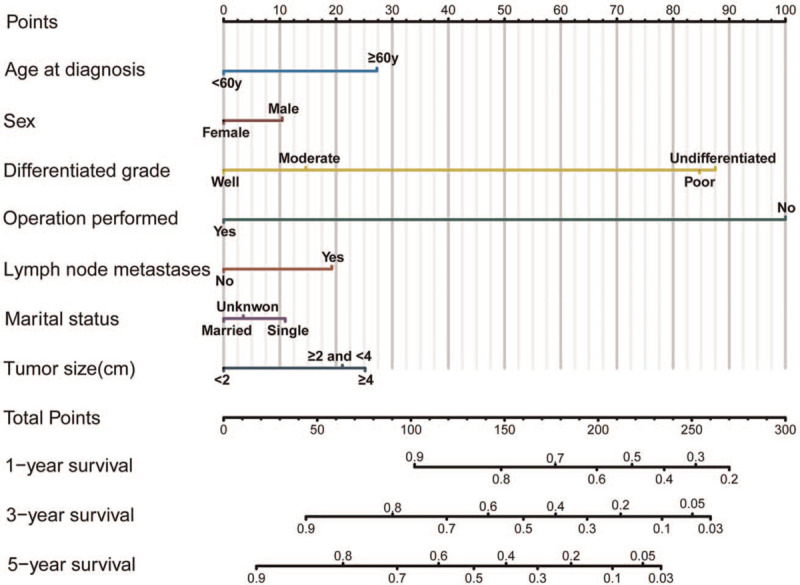
Nomogram predicting 1-, 3-, and 5-year survival in patients with metastatic nonfunctioning pancreatic neuroendocrine.

**Figure 2:**
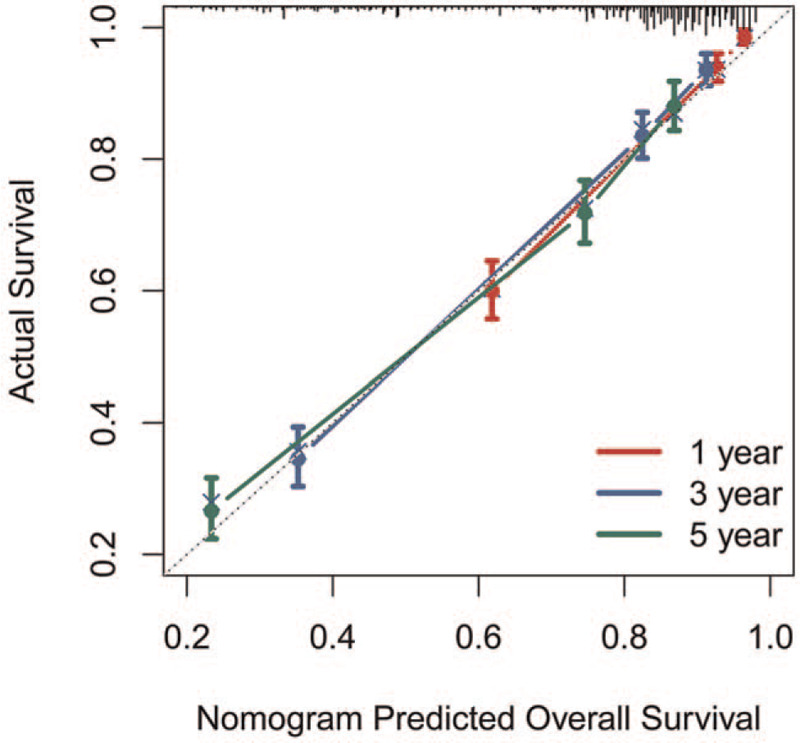
Calibration curve of the nomogram predicting 1-year(A), 3-year(B), and 5-year(C) OS in patients with metastatic nonfunctioning pancreatic neuroendocrine.

**Figure 3:**
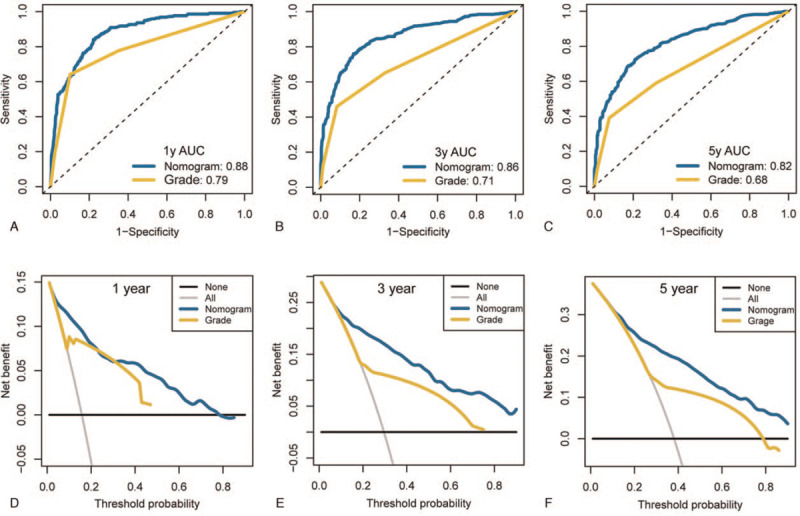
The discriminative ability, clinical usability and benefits of the nomogram compared to tumor differentiated grade. (A) The AUC of ROC curves for 1-year OS, (B) The AUC of ROC curves for 3-year OS, (C) The AUC of ROC curves for 5-year OS, (D) Decision curve analysis for 1-year OS, (E) Decision curve analysis for 3-year OS, (F) Decision curve analysis for 5-year OS.

Significant prognostic factors, including age at diagnosis, sex, tumor differentiation grade, lymph node metastases, surgical treatment, marital status, and tumor size, were incorporated into the nomogram to estimate 1-, 3-, and 5-year survival rates (Fig. 1). The calibration curves demonstrated excellent correlations between the nomogram prediction and actual observation (Fig. 2). The concordance index was 0.812, indicating the favorable discriminative ability of the predictive model. Furthermore, our model demonstrated better discriminative ability compared to tumor differentiated grade via ROC (1-year AUC: 0.88 vs 0.79, 3-year AUC: 0.86 vs. 0.71, 5-year AUC: 0.82 vs 0.68; Fig. 3 A-C).

DCA was conducted to compare the clinical usability and benefits of the nomogram with those of the tumor differentiated grade. As shown in Figure 3 D-E, the nomogram's 1-, 3-, and 5-year DCA curves exhibited larger net benefits across a range of death risks.

### 3.4 Statistical matching for surgical treatment

**Figure 4:**
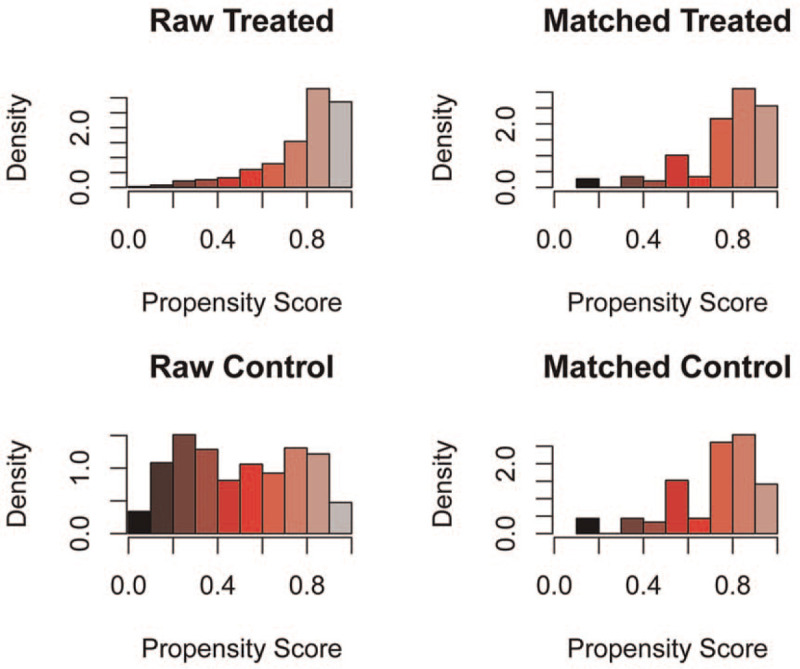
The histogram of raw data and matched data for surgical intervention. The histograms before matching was on the left while the histograms after matching was on the right. The similarity between treated and control group was related to the success of matching.

**Figure 5:**
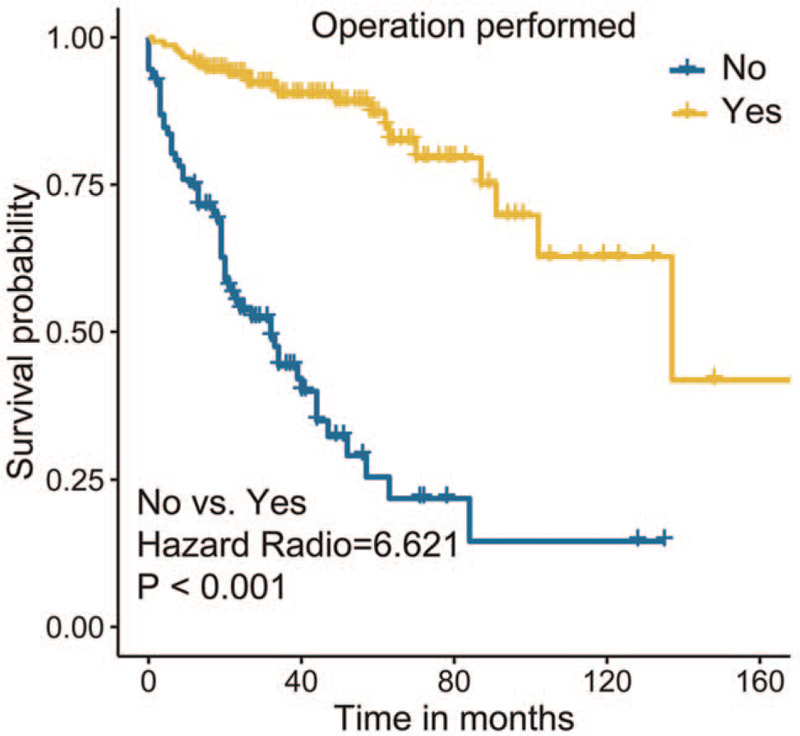
The survival curve of resection of primary site after CEM.

Statistical matching is an efficient method to lower the differences between groups on confounding variables and allows confidence in the strength of the observed outcomes of the study. To make the results more reliable, we performed CEM for surgical intervention to further assess its efficacy. As shown in Figure 4, the histograms after CEM were much more similar than the left-side histograms without matching, indicating that potential selection bias regarding the receipt of surgery was minimized. We then conducted Kaplan–Meier analysis for the new matched data, and found that resection of the primary site was associated with better survival in patients with metastatic NFpNET (no surgery vs surgery, hazard ratio = 6.621, *P* < .001; Fig. 5).

## 4 Discussion

A total of 1464 patients with metastatic NFpNETs were identified from the SEER database. Our study included 12 variables. Using the Cox regression model, we found that age, sex, tumor size, differentiated grade, lymph node metastases, resection of primary tumors, and marital status were significantly associated with the prognosis of metastatic NFpNET. Therefore, we used these significant prognostic factors to develop a nomogram for the estimation of 1-, 3-, and 5-year survival. Our model showed a high predictive accuracy and net benefits. As the largest sample of current research on metastatic NFpNET, our nomogram is helpful for decision making in clinical practice.

This is similar to previous studies showing that younger female patients were associated with a better prognosis in our study.^[7,8]^ The reason why females have a survival advantage is not clear, but may be attributed to the protective effect of estrogen on digestive tract tumors.^[9,10]^ The recent development of diagnostic techniques and treatments makes it possible for clinicians to diagnose pNET earlier and draw up a more appropriate treatment plan, which leads to superior survival in patients diagnosed in recent years.^[1]^ We also found that without lymph node metastases, patients with metastatic NFpNETs tended to be associated with superior outcomes. Some studies have also demonstrated the importance of nodal metastases.^[8,11]^ The staging standard of pNET by AJCC also includes lymph node metastases. There are quite a few articles that show that tumor size and differentiated grade are associated with survival time.^[12–15]^ This finding is consistent with our findings.

Whether primary tumor resection is conducive to metastatic NFpNET remains controversial.^[16,17]^ Capurso et al and Bettini et al reported that although the surgical group had a longer survival time than the non-surgical group, primary tumor resection was not significantly associated with survival of metastatic NFpNET patients. This may be due to the small sample size.^[18–20]^ In contrast, Keutgen et al and Franko et al demonstrated that primary tumor resection was significantly associated with improved survival in patients with metastatic NFpNET.^[21,22]^ Even patients who had a small-sized tumor (2 cm or smaller) need primary tumor resection.^[23]^ A previous study reported that primary tumor resection can alleviate tumor-related complications, such as left-sided portal hypertension, gastric outlet obstruction, gastrointestinal hemorrhage, or biliary obstruction.^[24]^ Liver metastasis occurs most frequently in patients with NFpNET. Givi et al reported that patients with liver metastases benefit only from the resection of primary neuroendocrine tumors.^[25]^ In our study, multivariate Cox proportional hazard models and CEM-based analyzes showed that resection of primary tumors was significantly associated with prolonged survival time of metastatic NFpNET patients in this cohort.

Previous studies have reported that chemotherapy plays an important role in the management of patients with advanced metastatic pancreatic neuroendocrine tumors, especially those with advanced unresectable and rapidly growing tumors.^[26,27]^ There are different chemotherapeutic regimens for well-differentiated and poorly differentiated tumors. Moreover, different chemotherapeutic drugs have different toxicities. However, the lack of detailed information in this regard in the SEER database limited our study. Therefore, the relationship between chemotherapy and survival of metastatic NFpNETs remains to be elucidated. Currently recognized effective radiotherapy methods, including peptide receptor radionuclide therapy (PRRT) with radiolabeled somatostatin analogs, have recently been utilized for NFpNET.^[28]^ Thus, radiotherapy methods may have a low impact on SEER research.

Marriage has been proven to have a protective effect on the prognosis of many cancers.^[29,30]^ We found that marriage was also associated with greater survival of metastatic NFpNETs on multivariable analyses. Li et al reported that unmarried patients lack the support they would have otherwise received from their partners and are likely to have unhealthy lifestyles (such as alcohol, tobacco consumption, drug abuse).^[29]^ In contrast, married patients can receive emotional support and social interaction.^[31]^ They also received more preventive healthcare and more aggressive treatments. The modern medical model reminds us that we must strengthen psychological and social support in patients with metastatic NFpNET.

However, this study had some limitations. First, unavoidable bias must exist in retrospective studies and cannot be completely eliminated, even with CEM. For the scientific nature of the research, large-scale randomized controlled trials should be applied in future research. Second, the SEER database lacks detailed information on adjuvant therapy, such as specific chemotherapy regimens and radiotherapy parameters. In addition, some clinical manifestations affecting prognosis, such as jaundice and stomach ache, are not available in this database. Lastly, the inclusion of patients diagnosed before 2010, the year of the latest WHO classification of gastroenteropancreatic NET, prevents the application of the distinction into the 3 categories G1-G2-G3, as the inclusion of different classifications may create bias about prognosis by itself. Therefore, the factors that may affect prognosis have not yet been included in our nomogram.

Despite these limitations, our study is the first to build a predictive model for metastatic NFpNET with the largest sample size, and also the first to use CEM to demonstrate the advantages of resection of primary tumors. Using ROC curve and DCA curve analysis, we found that the nomogram can clearly and accurately reflect the 1-, 3-, and 5-year OS rates of these patients. This predictive tool can help clinicians identify high-risk patients and more accurately assess patient survival times.

## Author contributions

**Conceptualization:** Lina Ge.

**Data curation:** Lina Ge, Haijin Li, Jiangang Zhao.

**Formal analysis:** Lina Ge, Liang Dong, Dengfu Peng.

**Investigation:** Guanmin Shang, Guoqi Tong.

**Methodology:** Weiying Wang.

**Resources:** Ying Li.

**Software:** Liping Qi.
